# Uncovering adaptive evolution in the human lineage

**DOI:** 10.1186/1471-2164-15-599

**Published:** 2014-07-16

**Authors:** Magdalena Gayà-Vidal, M Mar Albà

**Affiliations:** Evolutionary Genomics Group IMIM-UPF Research Programme on Biomedical Informatics, Barcelona Biomedical Research Park (PRBB), Aiguader 88, 08003 Barcelona, Spain; Institute of Biotechnology and Biomedicine (IBB), Universitat Autònoma de Barcelona (UAB), Barcelona, Spain; Catalan Institution for Research and Advanced Studies (ICREA), Barcelona, Spain

**Keywords:** Positive selection, Variation data, Divergence, Nervous system, Human evolution

## Abstract

**Background:**

The recent increase in human polymorphism data, together with the availability of genome sequences from several primate species, provides an unprecedented opportunity to investigate how natural selection has shaped human evolution.

**Results:**

We compared human branch-specific substitutions with variation data in the current human population to measure the impact of adaptive evolution on human protein coding genes. The use of single nucleotide polymorphisms (SNPs) with high derived allele frequencies (DAFs) minimized the influence of segregating slightly deleterious mutations and improved the estimation of the number of adaptive sites. Using DAF ≥ 60% we showed that the proportion of adaptive substitutions is 0.2% in the complete gene set. However, the percentage rose to 40% when we focused on genes that are specifically accelerated in the human branch with respect to the chimpanzee branch, or on genes that show signatures of adaptive selection at the codon level by the maximum likelihood based branch-site test. In general, neural genes are enriched in positive selection signatures. Genes with multiple lines of evidence of positive selection include taxilin beta, which is involved in motor nerve regeneration and syntabulin, and is required for the formation of new presynaptic boutons.

**Conclusions:**

We combined several methods to detect adaptive evolution in human coding sequences at a genome-wide level. The use of variation data, in addition to sequence divergence information, uncovered previously undetected positive selection signatures in neural genes.

**Electronic supplementary material:**

The online version of this article (doi:10.1186/1471-2164-15-599) contains supplementary material, which is available to authorized users.

## Background

The identification of signatures of positive selection in genes is a central theme in molecular evolution [1, 2]. The relative contribution of neutral and adaptive evolution is the subject of ongoing debate [1, 3–5]. A powerful approach to identify positive selection in coding sequences is the comparison of substitutions and polymorphic sites using the McDonald and Kreitman (MK) test [6]. This test builds on previously developed approaches to detect deviations from neutral evolution using divergence and polymorphism data [7, 8]. The MK test compares the number of non-synonymous (Dn) and synonymous (Ds) substitutions in a pair of species to the number of non-synonymous (Pn) and synonymous (Ps) polymorphisms in one of the species for the same coding sequence. Positively selected mutations reach fixation very rapidly, and thus increase non-synonymous divergence; however, they are rarely observed as polymorphic sites. As a result, if positive selection has played a significant role in the evolution of a gene, the ratio between non-synonymous and synonymous substitutions is expected to be larger than the ratio between non-synonymous and synonymous polymorphisms. The first ratio divided by the second ratio has been named the fixation index (FI) [9]. FI values larger than 1 indicate positive selection in the lineage of interest, and values lower than 1 indicate an excess of non-synonymous polymorphisms in the current population. Related statistics are the neutrality index (NI) [10], direction of selection (DoS) [11] and the proportion of adaptive substitutions (α) [12, 13].

Several authors have used the MK test to estimate the importance of positive selection in diverse genomes, with contrasting results depending on the species studied [5]. In most studies of humans, the estimated proportion of adaptive amino acid substitutions is very low [13–16]. One likely explanation is the presence of slightly deleterious segregating mutations caused by reduced effective population size, resulting in inflated non-synonymous SNPs relative to non-synonymous substitutions [17, 18]. Most deleterious mutations are expected to be present at low frequencies in the population; therefore, removing low frequency variants should improve the estimation of FI and other similar statistics [13, 19].

Other approaches to identify signatures of natural selection are based on the comparison of non-synonymous and synonymous substitutions in different branches of a tree obtained from a set of homologous sequences. Using data from several species has the advantage that we can target genes that show lineage-specific positive selection and discard genes that are also evolving under positive selection in related lineages, which is especially relevant when searching for species-specific traits. Genome-wide studies have shown that the pace of evolution of a protein often varies substantially in different branches of a tree [20–23], which is in agreement with the so-called over-dispersion of the molecular clock [24, 25]. Under lineage-specific positive selection, we expect an increase in the non-synonymous to synonymous substitution rate ratio in the corresponding branch. However, this pattern is also compatible with relaxation of the protein’s selective constraints.

Another widely used method to detect branch-specific natural selection patterns in the context of a phylogenetic tree is the branch-site-test [26]. This test is based on the comparison of the likelihood of a model in which all codons evolve neutrally or under purifying selection in all branches of the tree, with the likelihood of a model in which a fraction of the codons evolve adaptively but only in the branch of interest. This test and previous similar tests have been used by several authors to identify putative positively selected genes in humans [23, 27, 28]. Computer simulation studies indicated that the test tends to be conservative and that its power is limited when closely related species are compared [26, 29–31]. Statistically relevant comparisons with experimentally validated data are not yet available; however, Nozawa and colleagues have noted that known functional changes in vision genes are not always detected by the test [32].

Despite strong interest in understanding which evolutionary events have driven humans apart from their closest living relatives, our current knowledge of the associated molecular mechanisms remains very limited [33]. Recently, this question has started to be addressed using genome-wide approaches [23, 27, 28, 34–36]. Studies published to date have either considered human–chimpanzee divergence measures for the MK test, being unable to identify in which of the two species changes have occurred, or have only employed the branch-site test, missing the valuable information provided by polymorphism data.

Large-scale scans of positive selection have reported on various functional groups of genes enriched in positive selection signatures, including those involved in sensory perception [23], olfactory receptors [23, 28] and spermatogenesis [35]. Other gene-based studies have reported signatures of positive selection in several genes that affect brain size [33, 37, 38], including *ASPM* (abnormal spindle-like microcephaly associated), *CDK5RAP2* (*CDK5* regulatory subunit associated protein 2), *CENPJ* (centromere protein J) and *MCPH1* (microcephalin 1).

Here, for the first time, we used divergence and polymorphism data specific to the human branch that integrates the results of several divergence based approaches to identify positively selected genes. The analysis revealed that genes involved in nervous system functions are enriched in positive selection signatures in the human branch. We estimated that at least 40% of the non-synonymous substitutions in genes accelerated in the human branch, or in genes detected by the branch site test, have been adaptive. By combining results from the different tests we were able to identify several genes that show multiple lines of evidence of adaptive evolution in the 5–6 million years separating humans from chimpanzees.

## Results

### Effect of rare variants on the detection of positive selection

**Figure 1:**
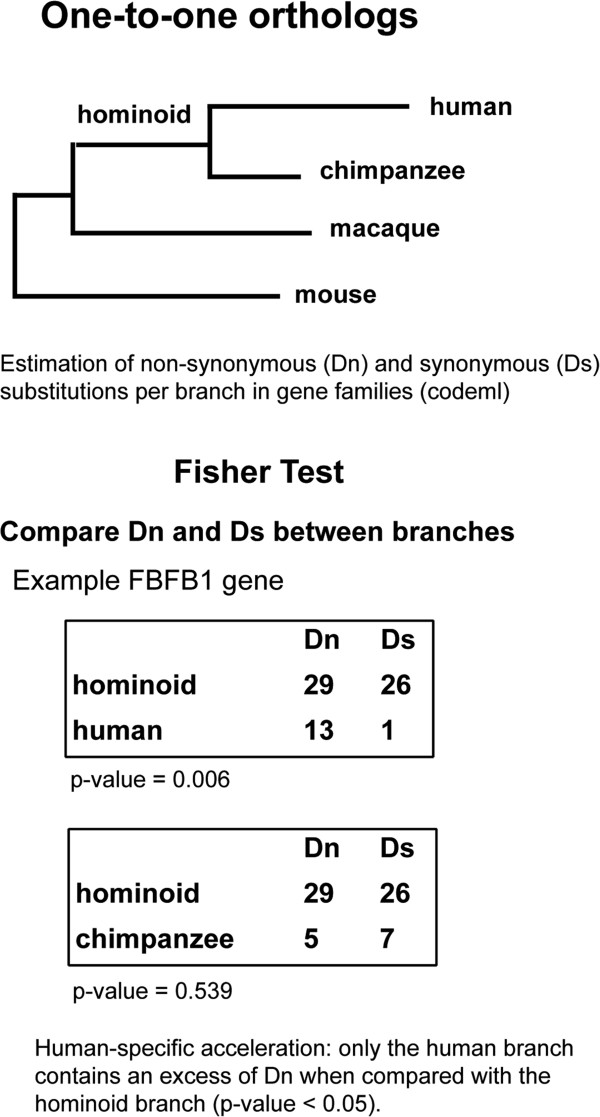
**One-to-one orthologous gene dataset and identification of human accelerated genes.** Fisher’s test using the estimated number of non-synonymous and synonymous substitutions in different branches of the tree. Data are for the Fas (*TNFRSF6*) binding factor 1 (*FBFB1*) gene.

**Figure 2:**
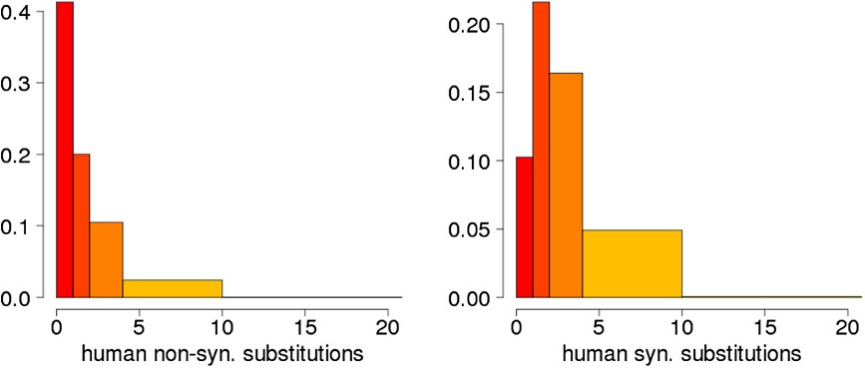
**Distribution of the number of non-synonymous (Dn) and synonymous (Ds) substitutions in the human branch.** For the sake of clarity, the x-axis has been cut at a value of 20. There are an additional 31 genes with more than 20 non-synonymous substitutions and 63 with more than 20 synonymous substitutions. The area of the bars is proportional to the number of genes. Equivalent bins in the two graphs are shown in the same color to facilitate the comparison.

To identify genes that are under positive selection in the human lineage we compared coding sequence divergence in the human branch after the split from the chimpanzee branch, representing 5–6 million years of human evolution, and polymorphism data from the current human population. We obtained gene families comprising one-to-one orthologs from human, chimpanzee, macaque and mouse from Ensembl Compara [39] (Figure 1). Subsequently, we estimated the number of Dn and Ds substitutions for each branch of the tree using the codeml maximum likelihood based program [40]. The application of several data quality controls (see Methods) resulted in 9,785 genes with human branch specific substitution data (Additional file 1: Tables S1 and S2). The average number of Dn and Ds per gene in the human lineage was 2 and 3.5, respectively (Figure 2). We mapped all available Pn and Ps substitutions from dbSNP (build 135) to the human coding sequences from our gene set. The vast majority (>95%) of the SNPs we employed had been identified in the 1000 Genomes Project [41]. Most genes (8,019) contained at least one polymorphic site, and about 35% of them had four or more polymorphic sites (Additional file 1: Table S3).

The FI for the complete gene set was 0.73. This value was obtained by summing the Dn, Ds, Pn and Ps values of individual genes (see Methods for details). The neutrality index (NI), which is 1/FI, was 1.37. The NI_TG_, a related index that takes into account gene heterogeneity [11], had a very similar value, 1.45.

The FI gradually increased as we considered higher frequency SNPs, reaching a value of 1 for SNPs with derived allele frequency (DAF) ≥60% (Table 1, and Additional file 1: Tables S4 to S7). This progression is consistent with the presence of slightly deleterious mutations in the population, because such mutations are expected to be comparatively less abundant at higher DAF values [13]. As expected, the proportion of adaptive substitutions (α) and other related statistics showed the same effect (Table 1). We observed a similar tendency when we used the minor allele frequency (MAF) instead of DAF (Additional file 1: Table S8). For SNPs with DAF ≥60%, we obtained α = 0.002, which corresponds to 0.2% adaptive substitutions.

**Table 1:** **Fixation index (FI) and related statistics for different human gene sets**

	SNPs DAF ≥ 1%				SNPs DAF ≥ 15%				SNPs DAF ≥ 30%				SNPs DAF ≥ 60%			
Data set	N	FI	DoS	α	N	FI	DoS	α	N	FI	DoS	α	N	FI	DoS	α
All genes	8019	0.73	-0.074	-0.361	5859	0.88	-0.031	-0.142	4744	0.94	-0.013	-0.059	2766	1.00	0.001	0.002
Human accelerated	182	1.60	0.116	0.375	15	1.55	0.109	0.356	136	1.69	0.131	0.409	75	1.68	0.129	0.405
Human BS test	216	1.16	0.037	0.137	172	1.27	0.058	0.209	135	1.39	0.080	0.278	78	1.66	0.123	0.398
Rapidly evolving	166	1.25	0.056	0.202	123	1.27	0.06	0.213	101	1.2	0.045	0.167	64	1.20	0.044	0.163
Mammalian-specific	77	0.71	-0.079	-0.413	57	0.95	-0.014	-0.058	47	1.04	0.010	0.042	28	0.91	-0.023	-0.100

Data are for genes with at least one polymorphic site. DoS: direction of selection; α: proportion of adaptive substitutions. SNP: single nucleotide polymorphism. DAF: derived allele frequency.

### Genes showing accelerated evolution in the human lineage

We identified genes that showed an excess of Dn substitutions when compared to the number of Ds substitutions in the human branch with respect to the hominoid ancestral branch (Figure 1, Fisher’s test p < 0.05). We only selected cases that passed a second test in which this same effect was not observed in the chimpanzee branch. Following this procedure, we obtained 198 genes showing human-specific acceleration (accelerated gene set, Additional file 1: Table S9 and S10). Taking all the genes together, we observed an FI of 1.6 in the human accelerated gene set, indicating a strong impact of positive selection in this group of genes. When we restricted the analysis to SNPs with DAF ≥60%, the overall FI of the accelerated genes increased to 1.68 and the proportion of adaptive substitutions reached 40.5% (Table 1).

**Figure 3:**
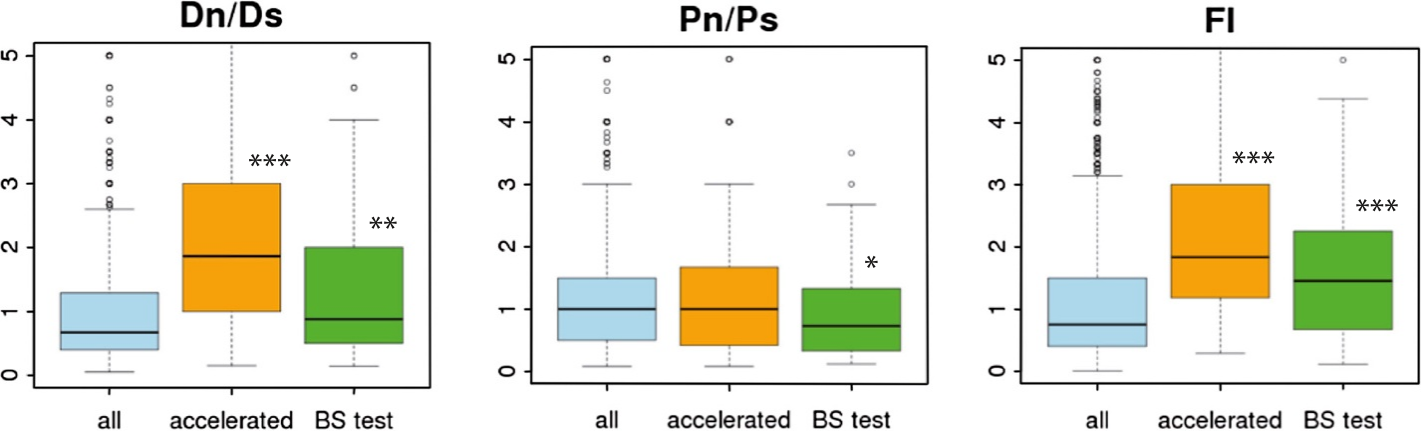
**Comparison of divergence and polymorphism data in different gene sets.** The data shown are for genes in which Dn, Ds, Pn and Ps ≠ 0 and Pn + Ps ≥ 4 (2,545 genes in the complete dataset; 109 human accelerated genes; 98 human branch-site (BS) test genes). The area within the box contains 50% of the data; the horizontal line is the median; small circles represent outliers (5%). The y-axis (FI) has been cut at FI = 5. We performed Mann–Whitney-Wilcoxon tests against the background (All genes) distribution: *p < 0.05; **p < 0.01; ***p < 0.001.

The analysis of the distribution of FI values in different genes confirmed important differences between human accelerated genes and the complete gene set (Figure 3, FI accelerated *vs.* all genes; Wilcoxon test p < 10^-5^). As expected, the Dn/Ds values in the human branch tended to be much larger in the accelerated gene set than in the complete gene set (Wilcoxon test p < 10^-5^), reflecting the accumulation of a larger number of fixed non-synonymous substitutions in the former set. In contrast, the distribution of Pn/Ps values was indistinguishable in the complete gene set and in the accelerated gene set (Wilcoxon test p = 0.91), indicating similar levels of selective constraints in both gene sets.

To better understand the effect of rapid gene evolution on the FI, when unlinked from human-specific evolutionary rate acceleration, we sampled from the complete set of genes a subset of genes that showed the same evolutionary rate distribution as the human accelerated genes, but which did not include any genes showing human-specific acceleration (rapidly evolving gene set, 198 genes, Additional file 1: Table S11). The FI value of the rapidly evolving gene set was 1.20 for SNPs with DAF ≥60% (Table 1), indicating that some of these genes are evolving adaptively. However, this value was much lower than the FI value of the human accelerated gene set (FI = 1.68), indicating that they are not so influenced by positive selection as the latter set. We also noted that the Pn/Ps ratio was higher in the set of rapidly evolving genes than in the human accelerated genes (1.06 *vs.* 0.88). This indicates that the former genes tend to evolve under more relaxed constraint than the latter ones, which is consistent with the fact that they are evolving rapidly not only in the human branch, but also in other branches.

### Genes detected by the maximum likelihood based branch-site test

The branch-site (BS) test is a maximum likelihood based test that compares the likelihood of an evolutionary model (H1) that includes branch-specific positive selection (some codons can evolve with ω > 1 in the branch of interest, but not in the rest of the branches) with the likelihood of a model (H0) that does not consider such a possibility (all codons are evolving with ω ≤ 1 in all branches) [26]. Several recent works have used this test to perform genome-wide scans of positive selection in genes from humans or other mammalian species [23, 27, 28, 42, 43]. Using this test, we identified 241 gene families that showed a signature of positive selection in the human branch at p < 0.05 (BS test set, Additional file 1: Table S12). A large number of these genes (124 genes, 51.4%) were also significant after false discovery rate correction (p < 0.05). The overlap with the accelerated gene set was relatively small (32 out of 241 genes, 13.3%).

The global FI of the human BS test gene set, considering all SNPs, was 1.16; however, this value increased to 1.66 for SNPs with DAF ≥60%, which corresponded to about 40% adaptive substitutions, similar to the values obtained for human accelerated genes (Table 1). The distribution of FI values in genes detected by the BS test set was skewed to values larger than 1 compared with the complete gene set (Figure 3, Wilcoxon test p < 10^-5^), but to a lesser extent than for the accelerated gene set. The Dn/Ds values were lower than for the human-specific accelerated genes, but still significantly higher than for the complete gene set (Wilcoxon test p < 0.01). Unexpectedly, the Pn/Ps values were lower in the BS test set than in the complete gene set (p = 0.027), indicating that genes selected by this test are biased towards highly constrained genes.

### Mammalian-specific genes

Another interesting set of genes to include in the comparison was phylogenetically restricted genes, many of which are likely to be important for lineage-specific adaptations [44]. Lineage-specific genes typically show higher than average non-synonymous to synonymous substitution rates [45–47], as well as intra-specific signatures of relaxed purifying selection [48]. However, it is still unclear if positive selection has significantly affected their evolution [49]. To answer this question, we crossed-checked a list of mammalian-specific genes extracted from the literature [50] with our set of orthologous genes from human, chimpanzee, macaque and mouse (mammalian-specific gene set, Additional file 1: Table S13). Both the Dn/Ds and Pn/Ps were about three times higher in this set than in the complete gene set (1.5 versus 0.55 for Dn/Ds, 2.14 *vs.* 0.77 for Pn/Ps); however, the FI, DoS and α were not affected (Table 1). This indicates that relaxation of purifying selection is the main driver of the rapid evolution of these genes.

### Adaptive evolution signatures in neural system genes

**Figure 4:**
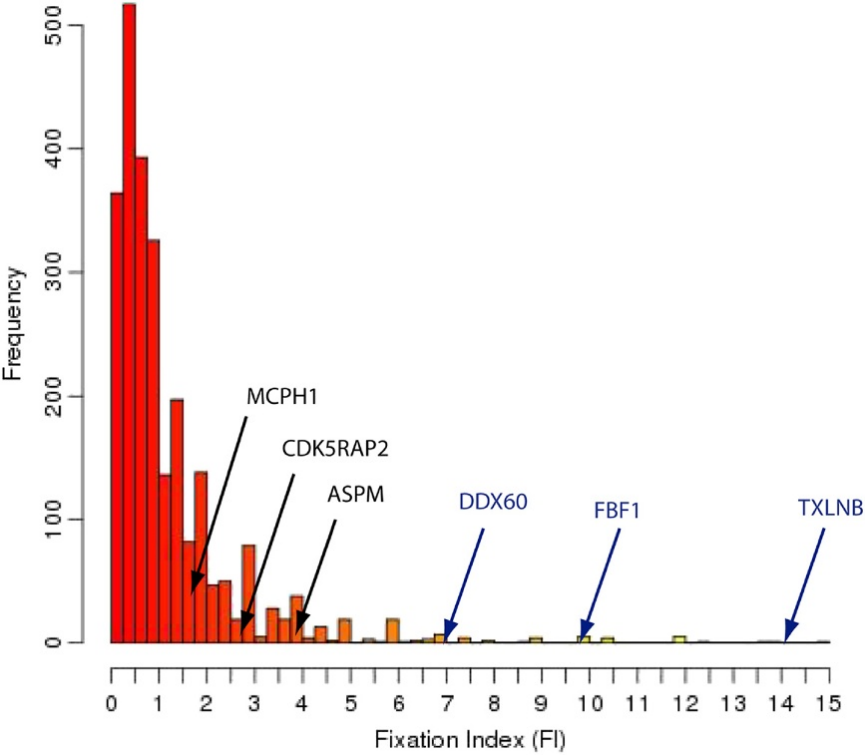
**Histogram of the distribution of the Fixation Index in human genes.** For the sake of FI estimation robustness, we selected genes in which Dn, Ds, Pn and Ps ≠ 0 and Pn + Ps ≥ 4. Genes with FI > 15 were not considered for the plot. The total number of genes included in the plot was 2528. Genes in blue are significant by the MK test (p < 0.05). *MCPH1* (ENSP00000342924) FI = 1.67; *CDK5RAP2* (ENSP00000343818) FI = 2.5; *ASPM* (ENSP00000356379) FI = 3.75; *DDX60* (ENSP00000377344) FI = 7; *FBF1* (ENSP00000401215) FI = 10.4; *TXLNB* (ENSP00000351206) FI = 14.

The comparison of divergence and polymorphism data for each gene can be used to identify putative positively selected genes [6]. To analyze the distribution of FI values, we focused on genes with at least four polymorphic sites (Figure 4). We used this threshold to avoid very unreliable FI estimates while maintaining a substantial number of genes for comparative analysis.

Applying the G test of independence described in the original MK test [6], we identified 26 genes with a positive selection signature at a p-value < 0.05. Five additional cases were identified when we also considered genes with a lower number of polymorphic sites (Additional file 1: Table S14). The genes in the list were involved in a variety of functions, including the immune response (e.g. *ISGF1* and *DDX60*), cellular adhesion (e.g. *CDC42EP1*, *FBF1*) and neural functions (e.g. *TXLNB*). *ASPM*, previously reported to be under positive selection in the human lineage [37], showed a high FI, but did not reach statistical significance in the MK test (p-value 0.094). This was not surprising when we considered that the FI of the significant genes was ≥ 7, whereas the FI of *ASMP* was 3.75. The position of some of these genes on the overall FI distribution is shown in Figure 4.

None of the genes that were marked as significant by the MK test passed a multiple test correction. This is probably a consequence of the closeness of the two species compared and the low diversity of the human population, both factors limiting the power of the test at the individual gene level. Nevertheless, seven of the genes were also identified by at least one divergence based method (human-specific accelerated evolution or the BS test), making them particularly strong candidates for human-specific adaptations (Table 2). Two of these genes are involved in neural development. The first is taxilin beta (*TXNLB*), encoding a protein that plays important roles in motor nerve regeneration [51], which was detected by all three methods. The second gene was syntabulin (*SYBU*), which mediates axonal transport and is required for the formation of new presynaptic boutons in developing neurons [52]. This gene was detected by the MK test and also displayed significant human-specific evolutionary rate acceleration.

**Table 2:** **Genes with multiple evidence of positive selection in the human lineage**

	Ensembl protein ID	Description	Dn	Ds	Pn	Ps	p-value MK	Divergence-based methods
*TLE6*	ENSP00000403096	Transducin-like enhancer of split 6	9	2	0	4	0.004	BS test
*USHBP1*	ENSP00000252597	Usher syndrome 1C binding protein 1	11	2	2	5	0.016	Accelerated
*SYBU*	ENSP00000415654	Syntabulin (syntaxin-interacting)	3	1	0	4	0.025	Accelerated
*C20orf96*	ENSP00000353470	Chromosome 20 ORF 96	11	2	1	3	0.04	BS test
*TXLNB*	ENSP00000351206	Taxilin beta	7	1	2	4	0.043	Accelerated, BS test
*TLE4*	ENSP00000365703	RTransducin-like enhancer of split 4	14	4	0	2	0.044	BS test
*FBF1*	ENSP00000401215	Fas (*TNFRSF6*) binding factor 1	13	1	5	4	0.044	Accelerated, BS test

MK: McDonald-Kreitman test; BS test: branch-site test; accelerated: showing human-specific evolutionary rate acceleration.

The other genes with multiple evidence of positive selection were Fas (*TNFRSF6*) binding factor 1 (*FBFB1*), which binds to keratin and plays a role in epithelial cell polarization [53]; the gene encoding Usher syndrome 1C binding protein 1 (*USHBP1*), which binds to the protein responsible for Usher syndrome type 1C, a sensory defect involving deafness and blindness [54]; and two genes from the Enhancer of split Groucho family, *TLE4* and *TLE6*, which are involved in cell fate decision and boundary formation [55].

**Figure 5:**
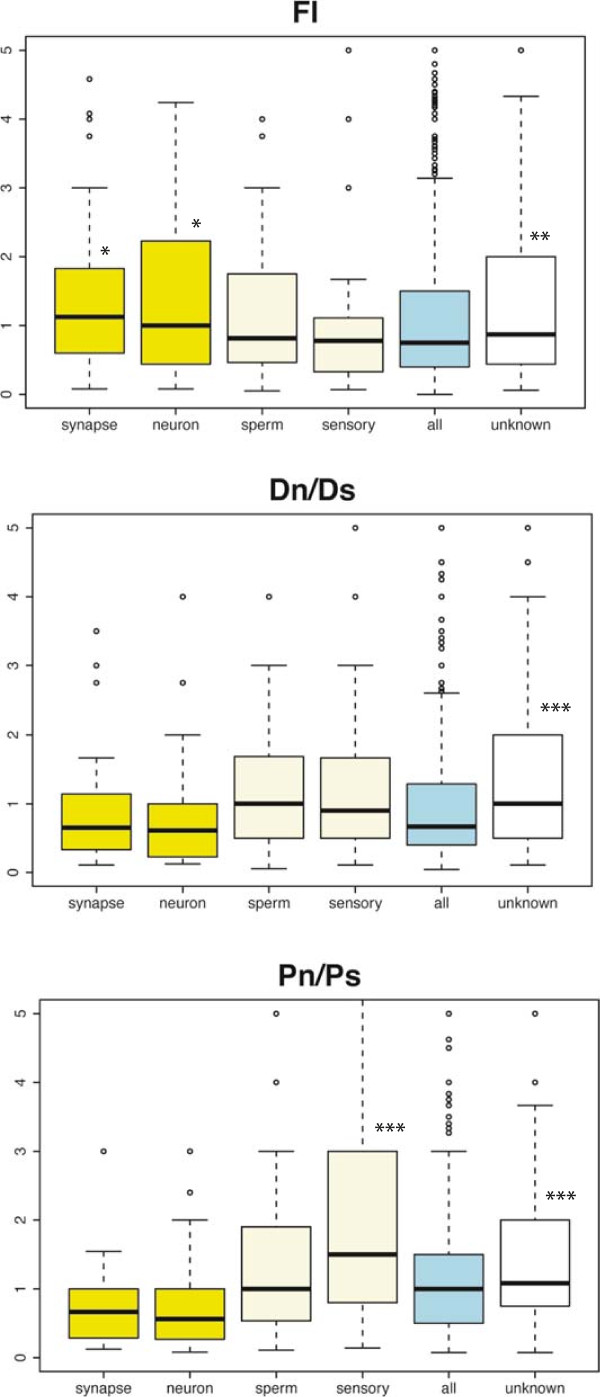
**Comparison of divergence and polymorphism data in different gene functional groups.** The set of genes is the same as in Figure 3. Gene Ontology (GO) annotations defined the functional groups. synapse: genes annotated under the “synapse” term; neuron: “neuronal cell body”; sperm: “spermatogenesis”; sensory: “response to stimulus”; all: complete gene set or background; unknown: genes with no annotated GO terms. The area within the box contains 50% of the data; the horizontal line is the median; small circles represent outliers (5%). The y-axis (FI) has been cut at FI = 5. We performed a Mann–Whitney-Wilcoxon test to assess if the differences with the background were statistically significant, for cases in which the median value in the gene set was larger than the background median: *p < 0.05; **p < 0.01; ***p < 0.001.

To further investigate the relationship between adaptive evolution and gene function, we examined the FI values of genes annotated under different Gene Ontology (GO) terms, focusing on terms with more than 40 annotated genes and including the group formed by genes with no associated GO terms (105 different groups, Additional file 1: Table S15). Among the 10 groups with the highest FI value, three presented a FI value distribution that was statistically different from the complete gene set or background (Wilcoxon test p < 0.05, Figure 5). Remarkably, two of these groups were related to nervous system functions: synapsis (42 genes, p = 0.029) and neuronal cell body GO terms (40 genes, p = 0.048). These two groups only shared eight genes, thus they were largely independent. The third significant group was genes of unknown function (712 genes lacking GO annotations, p = 0.0017). This indicates that many relevant genes for human evolution are probably uncharacterized. We also analyzed the data for two additional functional classes reported to be enriched in positively selected genes in previous genome-wide studies: spermatogenesis and response to stimulus [23, 28, 35]. The latter group included many genes involved in sensory perception, such as olfactory receptors. Both classes showed high Dn/Ds and Pn/Ps values, but their FI values were not significantly different from the background (Figure 5).

## Discussion

The comparison of the number of non-synonymous to synonymous changes in coding sequences is a powerful tool to uncover the action of natural selection. In the absence of selection we expect the number of non-synonymous substitutions per non-synonymous site (dN) and the number of synonymous substitutions per synonymous site (dS) to be similar. For the vast majority of protein coding sequences dN < dS, indicating that purifying selection prevents the fixation of deleterious mutations in coding sequences. A dN/dS ratio larger than 1 indicates positive selection and has been used in the past to identify putative positively selected genes using human and chimpanzee orthologous sequences [35]. However, many sites in a protein are subject to purifying selection, which undermines the detection of positive selection by this method. Additionally, a ratio larger than 1 is very rarely statistically significant for closely related species that have accumulated few substitutions since their separation. Increased sensitivity may be achieved by comparing divergence to polymorphism data, as in the MK test, or by comparing the non-synonymous to synonymous substitution ratio in different branches of the tree, either using complete sequence or codon-based divergence measurements. Here, we applied these different methods to a large set of mammalian gene families to gain a novel insight into the nature of human molecular adaptive processes.

Our overall estimate of the fraction of fixed adaptive substitutions (α) in the human lineage is very low, approximately 0.2%, which is consistent with previous studies [13–16]. In contrast, in species with much larger effective population sizes, such as mouse or fruit fly, the estimated α values are typically 40–60% [5]. One likely cause is the presence of segregating, slightly deleterious mutations in humans; for example, those caused by a recent population bottleneck, which will increase the number of Pn polymorphisms relative to the neutral expectation and downwardly bias the estimation of the FI and α value [17, 18]. The fact that the estimated α is higher for polymorphisms of increasing allele frequency in the population, as observed here and in other recent studies [19, 56], is consistent with this hypothesis. Another factor that may contribute to the low α in humans is the small population size itself. Species with small effective population sizes are expected to have a lower proportion of adaptive substitutions than species with a larger number of individuals, simply because the overall number of new mutations per generation is lower. Accordingly, a recent study based on 13 independent pairs of eukaryotic species showed that the rate of adaptive protein evolution is positively correlated with effective population size [57]. Finally, we used data derived from studies that employed individuals from different populations [41], which may have artificially increased the number of low frequency polymorphisms and decreased FI. Using SNPs with DAF ≥60% we obtained an overall FI of approximately 1 in the complete gene set. This value may be an underestimate of the real value if a portion of the deleterious mutations persists at such high frequencies [56, 58]. However, it is still useful to compare the relative contribution of positive selection in different selected subsets of genes.

In contrast to several earlier studies that used substitutions derived from human and chimpanzee sequence pairwise alignments for the MK test [34, 58], we employed human branch-specific substitutions, which means that we automatically discarded cases in which the signal of positive selection was in the chimpanzee branch and not the human branch. This was important to avoid mixing signals from both branches. Additionally, as the quality of the chimpanzee genome sequence is lower than that of the human genome, much of the signal when using both branches may be caused by false positives in the chimpanzee [59].

Branch-specific evolutionary rate acceleration can be caused by positive selection, decreased purifying selection, or a combination of both factors. As expected, we found strong evidence of positive selection in genes with accelerated evolution in humans. Surprisingly, we found no evidence of relaxed purifying selection in these genes, as they displayed similar Pn/Ps values than the rest of genes. Genes in the accelerated gene set showed remarkably high non-synonymous to synonymous substitutions; however, this alone was not sufficient to explain their high FI values, as deduced from the comparison with similarly rapidly evolving genes that were not human accelerated or with the set of rapidly evolving mammalian-specific genes. As shown here for humans, in bacteria, a correlation between species-specific protein evolutionary rate variation and positive selection signatures by the MK test was found [21].

The impact of positive selection on the BS test selected genes, as determined by the observed FI and α values, was similar or even lower than that in the accelerated genes. This was unexpected, because the BS test has been specifically designed to identify positive selection signatures [23, 26]. Given enough data, the test can be very useful to identify putatively positively selected sites, but it is also very sensitive to the presence of misaligned positions in the alignment, something that is difficult to correct for in large-scale analyses [59, 60]. Recently, we showed that, whereas the estimation of the ratio between non-synonymous and synonymous substitutions (dN/dS) is relatively robust to alignment errors, the results of the BS test are not [61]. Altogether, the detection of branches showing increased dN/dS appears to be a simple but effective method to detect positive selection. As polymorphism data become available for a larger number species, it will be possible to routinely combine the results of the tree-based methods (those that compare divergence in different branches) with the results of the MK test (those that compare divergence and polymorphism in a given branch), improving the detection of positive selection.

In humans, because of reduced diversity, the estimation of FI is only possible for some genes, and the power of the MK test is low. Long protein coding sequences tend to have more SNPs, which favors their detection by the MK test, compared with shorter coding sequences. Despite the important reduction in dataset size, focusing on the most informative genes (2545 genes with at least four polymorphic sites) allowed us to compare the gene FI distribution in 105 different functional classes, each containing at least 40 different genes. Under these conditions, three terms related to the nervous system were among the top 10 groups with the highest FI values; synapse, neuronal cell body and axon guidance, of which the first two showed a statistically significant deviation. Nervous system genes tend to evolve in a very constrained manner and few substitutions accumulate, even in cases in which the FI is larger than 1, which is likely to make their detection by the BS test difficult [22]. This might explain why they have not been detected in previous genome-wide scans using this test.

Functional groups found to be enriched in positive selection signatures in previous genome-wide studies using divergence data included sensory perception, immunity, spermatogenesis, apoptosis or regulation of transcription [23, 28, 35]. Among these functions, genes involved in regulation of transcription and spermatogenesis showed relatively elevated FI values in our study, although the differences with the background were not significant. The GO term ‘response to stimulus’ , containing many genes involved in sensory perception, showed abnormally high Pn/Ps ratios. This supports the hypothesis that these genes evolve under very low evolutionary constraints, as also found by [23]. Other possible explanations are that this set includes genes that have recently become pseudogenes, or that balancing selection in present-day populations is maintaining high non-synonymous polymorphism levels.

The size of the accelerated and BS test gene sets in our study (198 and 241 genes, respectively) was not sufficiently large to perform a systematic search for significantly overrepresented GO terms. Nevertheless, we counted how many genes were annotated under synapsis, neuronal cell body, response to stimulus and spermatogenesis in these gene sets. None of these classes achieved statistical significance, except synapse in the accelerated gene set, with five observed genes compared to one expected by chance (proportion test, p < 10^-3^). Taken together, our results strongly indicate that positive selection in neural genes is likely to have played a significant role in the human evolution. A study using human-macaque and mouse-rat comparisons reported that nervous system genes evolved in a more accelerated manner in primates than in rodents [62], also pointing to a link between increased non-synonymous substitutions in neural genes and primate evolution.

## Conclusions

Comparing the number of non-synonymous and synonymous substitutions in sister species can help identify sequences that have been the target of positive selection in one of the species. Here, we have combined divergence and variation measures to identify genes containing adaptive substitutions in the human lineage, but not in chimpanzees or the common ancestor or both species. The study uncovered an unexpected enrichment in positive selection signatures in neural genes.

## Methods

### Orthologous gene data sets

We obtained all available gene families containing one-to-one orthologous genes from human, chimpanzee, macaque and mouse from Compara at Ensembl (v.67) [39]. PRANK-F [63] was used to perform protein multiple sequence alignments, using the longest protein-coding transcript per gene. The following guide tree was specified: ((human: 0.01, Chimpanzee: 0.01): 0.03, macaque: 0.04, mouse: 0.32), adapted from [64]. Pal2Nal [65] was used to obtain the corresponding nucleotide coding sequence alignments. Ensembl Biomart [66] retrieved the GO terms. The set of orthologous protein families is available from Additional file 1: Table S1.

The set of mammalian-specific genes was obtained from [50]. These authors employed sequence similarity searches against a large number of genomes to classify genes according to their phylogenetic breadth. From their classification, we selected the group Mammalia from Additional file 1: Table S1 and used only the genes included in our study.

### Estimation of non-synonymous and synonymous substitutions

We estimated the number dN substitutions and the number of dS substitutions for each branch of the tree using the free ratio model of the codeml program in the PAML package [40]. Using this program, we also calculated the number of Dn substitutions and the number of Ds substitutions in each branch.

To improve the quality of the gene family sets, we only considered families in which all proteins were longer than 100 amino acids. We also discarded families showing dN/dS > 10 in any of the human, chimpanzee or hominoid ancestor branches, as such high dN/dS values may be caused by abnormally low number of synonymous substitutions and are not sufficiently reliable. The filtered dataset consisted of 9785 gene families (Additional file 1: Table S2). In the human branch, we estimated a total of 18936 non-synonymous and 34241 synonymous substitutions.

### SNP data

Human SNP data from the coding sequences of the 9785 genes were retrieved from UCSC (Common SNPs 135 build; GRCh37/hg19 assembly). SNPs included in the Common SNPs 135 build are characterized by a minor allele frequency (MAF) ≥ 1%. The SNPs are from different databases (including Applera, Hapmap, Complete Genomics and 1000 Genome Projects), the largest of which is the 1000 Genomes Project, which alone covers > 95% of the SNPs. The 1000 Genomes Project is based on genome sequencing reads from 1092 individuals from locations worldwide [41]. We obtained the number Pn and Ps polymorphisms for each coding sequence. 8019 of the 9785 genes were polymorphic in humans, including 14816 Pn and 19198 Ps SNPs (Additional file 1: Table S3). The fact that at present the ancestor allele is known for most SNPs allowed us to calculate the DAF, which is more informative of the gene’s evolutionary process than MAF. SNPs were classified into several categories (SNP sets) according to their derived allele frequency (DAF ≥ 1%, ≥ 15%, ≥ 30%, ≥ 60%; 1 ≤ DAF < 15%; 15 ≤ DAF < 30%; 30 ≤ DAF < 60%; 60 ≤ DAF < 100%).

### MK test and related statistics

Divergence and polymorphism data were compared using the MK test of neutrality by means of a G-test of independence with the Williams correction for continuity. The null hypothesis is that the proportion of amino acid replacements is independent of whether the changes are fixed or polymorphic [6].

The Fixation Index (FI) , Direction of Selection (DoS) and proportion of adaptive substitutions (α) (see Equations below) were calculated for each gene separately as follows:

Fixation Index:


Direction of Selection:


Proportion of adaptive substitutions:


where,

*Dn* is the estimated number of non-synonymous substitutions in the human branch

*Ds* is the estimated number of synonymous substitutions in the human branch

*Pn* is the observed number of non-synonymous polymorphic sites in humans

*Ps* is the observed number of synonymous polymorphic sites in humans

Values for sets of genes were obtained using the sum of the individual gene values of Dn, Ds, Pn and Ps. This is very similar to using a concatenated sequence alignment.

To explore how these statistics vary depending on the SNP set used, we employed i) SNPs with MAF ≥ 1%; ii) SNPs with MAF ≥ 15%; iii) SNPs exceeding certain DAF thresholds (DAF ≥ 1%, 15%, 30%, 60%); iv) SNPs belonging to a certain DAF interval category (1 ≤ DAF < 15%; 15 ≤ DAF < 30%; 30 ≤ DAF < 60%; 60 ≤ DAF < 100%); and v) SNPs belonging to a certain DAF category, but only considering genes with polymorphic sites (Table 1 and Additional file 1: Table S8).

For the comparison of individual gene FI distributions we selected genes in which Dn, Ds, Pn and Ps ≠ 0 and Pn + Ps ≥ 4, as this increased the robustness of the FI estimation. There were 2545 such genes. To compare the distributions we used the non-parametric Wilcoxon-Mann–Whitney test, as implemented in the R function wilcox.test. This test has been abbreviated as the Wilcoxon test in the manuscript.

### Identification of human branch-specific accelerated genes

To detect genes that showed evolutionary rate acceleration in the human branch, we assessed whether there was an excess of Dn substitutions over Ds substitutions in the human branch compared to the hominoid ancestor branch, but not in the chimpanzee branch compared to the hominoid ancestor branch, using Fisher's exact test (Figure 1). The expected tree topology of the selected cases was confirmed using the Neighbor-joining phylogenetic approach [67]. Only trees showing the expected tree topology were considered for subsequent analyses. The final accelerated gene set consisted of 198 genes (Additional file 1: Table S11).

A set of 198 control genes with a human dN/dS distribution similar to the human accelerated genes was obtained by sampling non-accelerated genes for different dN/dS value intervals. The dN/dS distribution of the two sets of genes did not show any statistical differences according to the Wilcoxon-Mann–Whitney test.

### Detection of positive selection using the branch-site test

We ran branch-site model A implemented in codeml on the sequence alignment data [26]. This test can detect positive selection even if it is only acting on a few sites in a specific lineage (the foreground branch) compared with the rest of the lineages (the background branches). In this study, the alternative hypothesis (positive selection) was compared to the null hypothesis (no positively selected sites) by means of a likelihood ratio test (LRT), because the two models are nested. The statistical significance was obtained with a chi-squared test using the R statistical package (http://www.r-project.org/).

The detection of positive selection by the branch-site test has been shown to be very sensitive to alignment quality [59, 60, 68, 69]. We manually inspected all cases in which there were more than 10 non-synonymous substitutions in the human branch, as such a large number of non-substitutions is atypical (Figure 2). We discarded all cases in which we detected misaligned positions caused by the presence of large gaps or the use of isoforms containing alternative exons.

## Electronic supplementary material

Additional file 1: Table S1: One-to-one orthologous gene families from human, chimpanzee, macaque and mouse included in the analysis. **Table S2.** Branch-specific non-synonymous (dN) and synonymous (dS) substitution rates from S1. **Table S3.** S1 human gene divergence and polymorphism data. **Table S4.** S3 DAF ≥ 1% taking into account only genes with at least one Pn or Ps. **Table S5.** S3 DAF ≥ 15% taking into account only genes with at least one Pn or Ps. **Table S6.** S3 DAF ≥ 30% taking into account only genes with at least one Pn or Ps. **Table S7.** S3 DAF ≥ 60% taking into account only genes with at least one Pn or Ps. **Table S8.** S1 human gene divergence and polymorphism data using different allele frequency intervals and McDonald-Kreitman test related statistics. **Table S9.** human branch-specific accelerated genes and Fisher’s test, dN and dS data. **Table S10.** S9 human gene divergence and polymorphism data. **Table S11.** 198 genes with similar human branch dN/dS distribution as S9. **Table S12.** 241 genes with significant positive selection in the human branch by the branch-site test (p < 0.05), and the corresponding false discovery rate p-value, divergence and polymorphism data. **Table S13.** mammalian-specific genes. **Table S14.** S1 genes that were significant by the McDonald-Kreitman test (p < 0.05). **Table S15.** Divergence and polymorphism summary data for genes annotated under different GO terms (N ≥ 40, at least four SNPs per gene). (XLS 9 MB)

## Abbreviations

BS: Branch site
DAF: Derived allele frequency
DoS: Direction of selection
FI: Fixation index
GO: Gene ontology
MAF: Minor allele frequency
MK: McDonald and Kreitman
NI: Neutrality index
SNPs: Single nucleotide polymorphisms.
